# Multiscale interactions of liquid, bubbles and solid phases in ultrasonic fields revealed by multiphysics modelling and ultrafast X-ray imaging

**DOI:** 10.1016/j.ultsonch.2022.106158

**Published:** 2022-09-06

**Authors:** Ling Qin, Kyriakos Porfyrakis, Iakovos Tzanakis, Nicole Grobert, Dmitry G. Eskin, Kamel Fezzaa, Jiawei Mi

**Affiliations:** a School of Engineering, University of Hull, Hull HU6 7RX, UK; b Faculty of Engineering and Science, University of Greenwich, Kent ME4 4TB, UK; c Department of Mechanical Engineering and Mathematical Sciences, Oxford Brookes University, Oxford OX3 0BP, UK; d Department of Materials, University of Oxford, Oxford OX1 3PH, UK; e Brunel Centre for Advanced Solidification Technology, Brunel University London, Uxbridge UB8 3PH, UK; f Williams Advanced Engineering, Grove OX12 0DQ, UK; g The Advanced Photon Source, Argonne National Laboratory, Argonne 60439, USA

**Keywords:** Multiphysics modelling, Ultrasonic bubble dynamics, Ultrasound materials processing, Liquid-bubble–solid interaction, Synchrotron X-ray imaging

## Abstract

The volume of fluid (VOF) and continuous surface force (CSF) methods were used to develop a bubble dynamics model for the simulation of bubble oscillation and implosion dynamics under ultrasound. The model was calibrated and validated by the X-ray image data acquired by ultrafast synchrotron X-ray. Coupled bubble interactions with bulk graphite and freely moving particles were also simulated based on the validated model. Simulation and experiments quantified the surface instability developed along the bubble surface under the influence of ultrasound pressure fields. Once the surface instability exceeds a certain amplitude, bubble implosion occurs, creating shock waves and highly deformed, irregular gas-liquid boundaries and smaller bubble fragments. Bubble implosion can produce cyclic impulsive stresses sufficient enough to cause µs fatigue exfoliation of graphite layers. Bubble-particle interaction simulations reveal the underlying mechanisms for efficient particle dispersion or particle wrapping which are all strongly related to the oscillation dynamics of the bubbles and the particle surface properties.

## Highlights

• The volume of fluid (VOF) and continuous surface force (CSF) methods were used to develop abubble dynamic model for the simulation of a single bubble oscillation, implosion behaviour and the coalescence of two bubbles under ultrasound.
• Simulations of bubble-bulk material interaction reveal that the shock wave at bubble implosion can produce sufficient stresses to cause microsecond fatigue exfoliation of graphite layers.
Simulation of bubble-particle interaction indicates that hydrophobic particles tend to attach to the bubble boundary, causing asymmetric bubble shape development and possible particle wrapping to occur.

## 1 Introduction

In a liquid flow containing moving bubbles and solid phases, the dynamic interactions among the liquid, bubbles and solid phases are multiphysics complex phenomena and sometimes highly transient. They are commonly found in the flowing water in rivers, lakes and sea [1], [2], in ultrasound cleaning and medical treatments [3], in sonoprocessing of materials [4], [5], etc. Quite often the bubbles and solid phases vary in size, as well as in physical, chemical, mechanical or biological properties [6]. Hence, the interactions often occur in multilength (nm to mm) and multi-time (ns to minutes) scale. Research on the bubble dynamics and the liquid-bubble-solid dynamic interactions has been the central theme of fluid dynamics research for many years. Recently, the effects of applying an alternating acoustic pressure field on a complex liquid-bubble-solid system have attracted much attention in the research community [7]. For example, Leighton et al. [8] and Ma et al. [9] observed the movement of a single bubble under an ultrasound wave, and found that the shape and state of the bubble is closely linked to the acoustic pressure. Kim et al. [10] did similar but more systematic work, and pointed out that the shape, oscillation and splitting of bubbles are indeed dependent on the initial bubble size and the amplitude of the pressure wave. Versluis et al. [11] also reported that a moving bubble may oscillate asymmetrically under a certain frequency range. In addition to pressure, the solid materials and their surface properties also have impact on the bubble dynamic behaviours. Shima et al. [12] reported the migratory behaviours of bubbles when they approached and collapsed at a compliant surface. In this direction, laser-induced cavitation bubble was often used because an isolated single bubble can be easily produced nearby a solid surface for studying the interaction. For example when a bubble is approaching a flexible membrane [13], [14], a composite surface [14], an elastic boundary [15], a flat rigid surface [16], or a flat free surface [17]. Another important direction is to investigate or simulate how a deformable solid structure responds to the impulsive pressure/stresses induced by an oscillating or imploding bubble. Duncan et al. [18], [19] did such simulation by considering a compliant membrane as a simple spring. Their results agreed well with the experiments by Shima et al. [12]. Chahine et al. [20] developed a 3D bubble dynamics model using the finite-element method (FEM). They simulated the interaction between a free-floating surface piercing object and an exploding bubble. Klaseboer et al. [21] also simulated an underwater exploding bubble and its interaction with a flat plate numerically using the FEM and boundary-element method (BEM). However, the FEM and BEM methods have inherent limitations when simulating high viscous fluids, vortices and tracking the evolution of interfacial boundary [22]. In particular, those modelling and simulation work generally lack calibration and validation by experiments. Hence, many aspects of the fundamental issues and underlying physics of these highly complex dynamic processes have not been fully understood.

Conventionally, highspeed optical imaging has been widely used in observing bubble dynamic behaviours [23]. However, there are inherent problems due to light absorption, reflection, and multiple scattering at the particle or bubble boundaries. Since 2011, our group has carried out extensive studies on the dynamics of ultrasonic bubbles and acoustic flow in different liquid media and their effects on the solid–liquid interface [24], solid phase during alloy solidification [25] as well as graphite layer exfoliation dynamics [26]. We have used the ultrafast synchrotron X-ray imaging facility (up to 271,554 fps) available at the sector 32-ID-B of the Advanced Photon Source (APS) for the above studies. With high spatial (∼2 µm) and temporal resolution (sub µs), the ultrafast synchrotron X-ray phase-contrast imaging (PCI) can “see” through liquid media, bubbles, and particles [27], [28], [29] and it is an ideal tool for capturing the dynamic movement or behaviours across particle and bubble boundaries [30].

In this work, a multiphysics numerical model was developed to simulate single bubble oscillation and implosion dynamics. For bubble boundary evolution simulation, the use of a robust, accurate and computational efficient interface tracking technique is essential [31]. The volume of fluid (VOF) is a classical, simple and well-adopted robust numerical method for handling topological evolution of an interface in 2D and 3D space [32]. The VOF is much more computationally efficient compared to other techniques, e.g., the moving grid, the level set and phase field method. To account for the surface tension effect of the bubble boundary, a continuum surface force (CSF) model was used. The volume force due to surface tension is applied onto the fluid elements in a finite thickness transition region [33]. Here, we used the VOF method in conjunction with the CSF method for the modelling work. Such combined numerical approach has been tested successfully by Tomiyama et al. [34] and Zu et al. [35] in their work of simulating the growth, oscillation and implosion of bubbles in different flow conditions. Furthermore, coupled bubble-bulk graphite materials interaction and bubble-particle interaction simulation were also made based on the validated models, providing much more insight on understanding more quantitatively the mechanisms of microsecond fatigue exfoliation of graphite layer and the bubble-induced particle dispersion dynamics and particle wrapping dynamics.

## 2 Mathematical formulation and numerical methods

### 2.1 The governing equations

#### 2.1.1 The continuity equations

In this work, the liquid and gas phases are treated as immiscible fluids with no slip between them, the continuity equation for the liquid and gas phase are [36]:

(1) $$\frac{\partial \left(\alpha_{l}\rho_{l}\right)}{\partial t}+\nabla \left(\alpha_{l}\rho_{l}U\right)=0$$

(2) $$\frac{\partial \left(\alpha_{g}\rho_{g}\right)}{\partial t}+\nabla \left(\alpha_{g}\rho_{g}U\right)=0$$

where $\rho_{l}$ and $\rho_{g}$ are the liquid and gas density respectively; $\alpha_{l}$ and $\alpha_{g}$ are the volume fraction of the liquid and gas phase, respectively, with the restriction of $\alpha_{l}+\alpha_{g}=1$. $\alpha_{l}=1$ denotes the liquid phase and $\alpha_{g}=1$ denotes the gas phase; U is the averaged velocity of the two phase flow.

The VOF method is a simple and economical way for tracking free boundaries [37]. $\alpha_{l}$ represents the volume fraction of the liquid, and thus the volume fraction of the gas is $\alpha_{g}=1-\alpha_{l}$. In the interface region, α varies from zero to unity.

Sum of the continuity equations for each phase leads to the overall continuity equation [38]:

(3) $$\frac{\partial \rho }{\partial t}+\nabla \cdot \left(\rho U\right)=0$$

where $\rho =\rho_{l}\alpha_{l}+\rho_{g}\alpha_{g}$ and is the mixed density of the gas and liquid phase flow.

#### 2.1.2 The momentum equation

The momentum equation is [39]:

(4) $$\frac{\partial }{\partial t}\left(\rho U\right)+\nabla \cdot \left(\rho UU\right)=-\nabla p+\nabla \cdot \tau +\rho g+\sigma k\nabla \alpha_{l}+F_{a}$$

where p is the pressure; g is the acceleration of gravity; σ is the surface tension coefficient; $F_{a}$ is the force generated at the sonotrode wave emitting surface and is described in detail in Eq. (12). The term,$\sigma k\nabla \alpha_{l}$ on the right hand of Eq. (4) presents the effect of surface tension force acting on the interface between the gas and liquid phase base on CSF method [33], [40]; k is the interface curvature and is calculated by:

(5) $$k=-\nabla \cdot \left(\frac{{\tilde{\alpha }}_{l}}{\left|{\tilde{\alpha }}_{l}\right|}\right)$$

where ${\tilde{\alpha }}_{l}$ is obtained from the volume fraction $\alpha_{l}$ by smoothing it over a finite region along the interface using the Lafaurie filter [41]. $\left|{\tilde{\alpha }}_{l}\right|$ is the absolute value of ${\tilde{\alpha }}_{l}$. More detailed descriptions of the momentum equation are given by Yin et al. [42], τ is the viscous stress tensor of Newtonian fluid and satisfies the relation below:

(6) $$\tau =\mu \left(\nabla U+{\left(\nabla U\right)}^{T}-\frac{2}{3}\left(\nabla \cdot U\right)I\right)$$

where I is the unit tensor,$\mu =\mu_{l}\alpha_{l}+\mu_{g}\alpha_{g}$ is the average dynamic viscosity.

#### 2.1.3 The energy equation

The energy equation expressed in terms of temperature T is written as:

(7) $$\begin{array}{c} \left[\frac{\partial \left(\rho T\right)}{\partial t}+\nabla \cdot \left(\rho TU\right)\right]+\left(\frac{\alpha_{l}}{\Omega_{l}}+\frac{\alpha_{g}}{\Omega_{g}}\right)\left[\frac{\partial \left(\rho K\right)}{\partial t}+\nabla \cdot \left(\rho KU\right)\right]\\ =\left(\frac{\alpha_{l}}{\Omega_{l}}+\frac{\alpha_{g}}{\Omega_{g}}\right)\left[\frac{\partial p}{\partial t}+\nabla \cdot \left(\tau \cdot U\right)\right]+\left(\frac{\alpha_{l}\lambda_{l}}{\Omega_{l}}+\frac{\alpha_{g}\lambda_{g}}{\Omega_{g}}\right)\left(\nabla^{2}T\right) \end{array}$$

where $\Omega_{l}$ and $\Omega_{g}$ are the heat capacity of the liquid and gas phases respectively at a constant pressure;$K=U^{2}/2$ is the kinetic energy; ∇·(τ·U) is the shear stress on the flow [43]; $\lambda_{l}$ and $\lambda_{g}$ is the thermal conductivity of the liquid and gas phase, respectively.

For the liquid phase, the Tait equation of state was used [44]:

(8) $$p=\frac{\rho_{0}c_{l}^{2}}{n}\left({\left(\frac{\rho }{\rho_{l}}\right)}^{n}-1\right)+p_{0}$$

where, $\rho_{0}$ = 998.2 kg/m^3^ is liquid (water) density at the reference pressure of $p_{0}$ = 3490 Pa. $c_{l}$ the speed of sound in liquid; the exponentn = 7.15 was used due to the weakly compressibility of Di-ionised (DI) water [45]. For the gas phase, a polytropic equation of state was used:

(9) $$p=\chi \rho_{g}^{\gamma }$$

where,χ = 0.12 kg/m^3^ is a constant calculated with ideal gas at 298 K and an ambient pressure of 10320 Pa [46]; the exponent γ is dependent on the thermodynamic process inside the bubble. In an isothermal process, it is unity. In our case, γ= 1.04 was chosen.

### 2.2 The fluid-bubble-solid interaction simulation

#### 2.2.1 The geometry and mesh structures

**Fig. 1:**
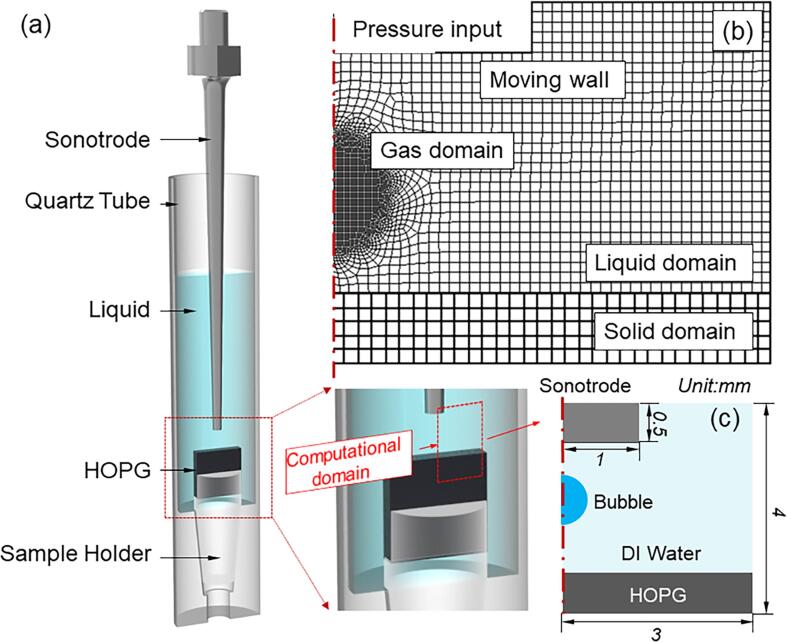
(a) A CAD rendering, showing the HOPG sample arrangement in the ultrasound liquid phase exfoliation experiment, (b) A 2D sectional view of the computational domain (mesh structures) based on the geometry above the HOPG, including the liquid, HOPG solid and bubble domain as well as their boundary conditions; (c) an overview of model geometry.

In this model, the solid used is the Highly Oriented Pyrolytic Graphite (HOPG, 10 × 10 × 2 mm from Agar Scientific ltd). Its boundary was set as a hard elastic surface for studying its interaction with an incoming bubble at implosion. The bubble dynamics model and the fluid–solid interaction model were coupled together to achieve such simulation. Fig. 1 shows the computational domains and the meshes. Square meshes were used in the liquid, solid and bubble interior domains. Gradiently-refined tetrahedral meshes were used near the bubble boundary. By tailoring the mesh parameters, we made the aspect ratio, jacobian ratio, mesh metric, and orthogonal quality close to or above 0.9, and the parallel deviation close to 0 as listed in Table 1. In such combination, the quality of the meshes was sufficient for subsequent numerical computing.

**Table 1:** Optimised mesh parameters.

Parameters	Mesh@Fig. 1	Mesh@Fig. 2
Aspect ratio	0.92	0.89
Parallel Deviation	0.04	0.07
Jacobian Ratio	0.98	0.94
Mesh Metric	0.96	0.89
Orthogonal Quality	0.94	0.91

#### 2.2.2 The boundary, initial conditions and materials properties

The sonotrode ultrasound radiating surface was set as a moving wall, vibrating with the velocity [46] of:

(10) $$V\left(t,y\right)=V_{0}\sin\left(\omega t\right)\cos\left(\varepsilon y\right)$$

here $V_{0}$ is defined as:

(11) $$V_{0}=\frac{p_{a}}{\rho_{l}c_{g}}$$

where ω=2πf is the angular frequency and f is the frequency; $\varepsilon =\omega /c_{l}$ is the wave number of acoustic wave, in this work, an ultrasound processor with a fixed frequency of 30 kHz (Hielscher UP100H) was used；$p_{a}$ is the pressure amplitude. The real time X-ray images can be used to measure the amplitude, A, in DI water. Therefore, the corresponding $p_{a}$ can be calculated by $p_{a}=A\rho c\omega$. Furthermore,$F_{a}$ described in Eq.(4) is the mean force per unit due to the ultrasound wave. In this case, $F_{a}$ is defined as [47]:

(12) $$F_{a}=\frac{p_{a}^{2}}{\rho_{l}c_{l}^{2}}\left(\frac{1}{2}-\cos\omega t\right)\left(\varepsilon \sin2\varepsilon y\right)$$

where $c_{l}$ is the speed of sound in liquid;y is the vertical distance away from the wave origin in the y-axis direction. Eq. (12) was included in the moving wall boundary condition by using a User-Defined Function (UDF). The bottom and two sides of the quartz tube were defined as stationary rigid walls. The pressure amplitude, liquid properties and boundary conditions are listed in Table 2.

**Table 2:** Properties used for bubble dynamics simulation [48], [49].

Parameters	Symbol & Unit	DI water	Gas bubble
Sound speed	$C(m.s^{-1})$	1482	
Surface tension	$\sigma (N.m^{-1})$	0.0725	
Dynamic viscosity	μ(Pa.s)	9.982 × 10^−4^	1.589 × 10^−5^
Density	$\rho (kg.m^{-3})$	998.2	1.0
Thermal conductivity	λ(W/(m.K))	0.677	0.026
Heat capacity	Ω(J/(kg.K))	4220	1000
Driven Pressure	$p_{a}\left(MPa\right)$	1.5	

Firstly, a steady-state pressure field without bubbles was calculated, and then the patch method [50] was used to seed a spherical bubble into the computational domain in a region near the top of the HOPG sheet. The seed bubble was placed when the sonotrode tip moved up (the rarefaction part of the acoustic cycle). The initial bubble radius ($R_{0}$=150 μm) was determined from the X-ray images as described in section 3.1. According to the X-ray images, the edge of the cavitation zone in the DI water is about 1.2 mm away from the sonotrode tip. Hence, we set the bubble center at 1 mm down from the tip surface to ensure that the bubble was in the cavitation zone. The internal pressure of the initial bubble ($p_{i}$= ∼14000 Pa) was chosen to make the simulated bubble radius match approximately the observed bubble radius at the recorded time steps in the X-ray images.

#### 2.2.3 Coupling of the bubble dynamics and fluid–solid interaction

Firstly, Eq. (1) to Eq. (9) were all solved to obtain the pressure and velocity fields induced by bubble implosion based on the Navier-Stokes (N-S) equation in all domains (gas, liquid and solid phase). The solid phase domain is handled as a porous media , which means that the flow is ignored because the damping in this region is significant. The most importantly information is the pressure distribution/fluctuations caused by the shockwave produced at bubble implosion, and the associated velocity fields due to resulting bubble microjet and acoustic streaming flow action onto the solid phase domain.

Secondly, the pressure distribution $\left\{\text{p}\right\}$ in the solid phase was exported from ANSYS Fluent model and mapped to another FEM based Fluid-Structure Interaction (FSI) model in ANSYS Mechanical as the load boundary conditions in Eq. (13) and Eq. (14). The FEM model was used to calculate the $\left\{\nu \right\}$ and $\left\{\ddot{\nu }\right\}$ of the HOPG sheet subjected to the pressure and gravity loads. The governing equations for anisotropic, linear-elastic solid are as below [51]:

(13) $$\left[M_{s}\right]\left\{\overset{¨}{\nu }\right\}+\left[N_{s}\right]\left\{\nu \right\}=\left[F_{k}\right]+\left[\varphi \right]\left\{p\right\}$$

(14) $$\left[\begin{array}{c} M_{s}\;\text{0}\;\\ \rho \varphi^{\text{T}}\;{\text{M}}_{\text{f}} \end{array}\right]\left\{\frac{\overset{¨}{\nu }}{\overset{¨}{p}}\right\}+\left[\begin{array}{c} N_{s}\;\varphi \;\\ 0\;{\text{N}}_{\text{f}} \end{array}\right]\left\{\frac{\nu }{p}\right\}=\frac{F_{k}}{F_{f}}$$

where $\left\{\nu \right\}$ and $\left\{\ddot{\nu }\right\}$ are the nodal displacement and acceleration vectors, respectively. $\left[M_{s}\right]$ is the structural mass matrix; $\left[M_{f}\right]$ is the fluid mass matrix;$\left[N_{s}\right]$ and $\left[N_{f}\right]$ are the structural and fluid stiffness matrix; $\left[F_{k}\right]$ and $\left[F_{f}\right]$ are the structural and fluid force matrix, and $\left[\varphi \right]$ is a coupling matrix that represents the effective surface area associated with each node in the fluid–structure interface. The graphite properties used for fluid–solid interaction simulation are listed in Table 3.

**Table 3:** Properties used for fluid–solid interaction simulation [52].

Parameters	Symbol & unit	Graphite
Compressive strength	$\sigma_{c}$(MPa)	30.8
Tensile strength	$\sigma_{t}$(MPa)	8.3
Young's modulus	G(GPa)	6.6

#### 2.2.4 Numerical methods and computing hardware

For Eq. (4) and Eq. (7), the SIMPLE algorithm [53] was used for pressure–velocity coupling. The pressure staggering option (PRESTO!) [54] scheme was used for discretization of the pressure, a second-order upwind scheme [55] was used for momentum discretization in Eq. (4) and energy discretization in Eq. (7). A compressive scheme [56] was used for volume fraction discretization in Eq. (3). The simulations were computed in double precision with a segregated solver. The time step was set between 1e^−7^ s ∼ 1e^−10^ s. The simulations were made by using the commercial software package ANSYS Fluent 19.0 in the computing node (C183, with 28 cores and 256 GB of RAM) of the Viper High Performance Computing (HPC) cluster in the University of Hull. Each case of simulation took approximately 54 h to complete.

### 2.3 The bubble-particle interaction simulation

#### 2.3.1 The governing equations

In this study, we simplify the movement of the solid particles, only considering a single solid particle moving in the vertical direction, and its interaction with an oscillating bubble, thus, the equation of particle motion can be written as:

(15) $$m_{p}\frac{\mathrm{du}}{\mathrm{dt}}=F_{f}+F_{s}-F_{g}$$

where u is the particle velocity and $m_{p}$ is the particle mass.$F_{f}$ is the fluid force acting on the particle surface; which can be calculated by:

(16) $$F_{f}=\iint_{A_{P}}-pn_{s}dA+\iint_{A_{P}}\mu \left(\nabla U+\nabla U^{T}\right)∙n_{s}dA$$

The two terms at the right side correspond to the integrals of pressure stress and viscous stress over the entire particle surface area $A_{P}$, which can be computed with the equations of fluid flow.

$F_{s}$ is the surface tension arising due to contact at the bubble interface. It is computed with Eq. (17), which was proposed by Kintea et al.[57].

(17) $$F_{s}=\int_{\mathrm{CL}}\sigma \varepsilon dl=\iint_{A_{P}}\left|\nabla_{S}\alpha \right|\sigma \varepsilon dA$$

where $\nabla_{s}\alpha$ is the surface gradient of the liquid volume fraction at the particle surface. $F_{g}=m_{p}g$ is the particle gravity.

#### 2.3.2 The geometry and mesh structures

**Fig. 2:**
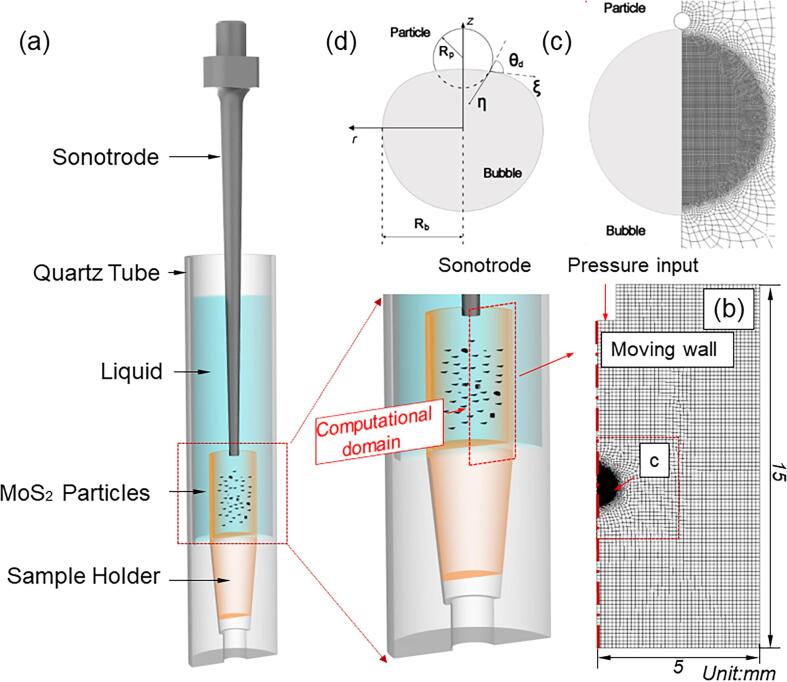
(a) A CAD rendering (including the enlarged view on the right), showing the MoS_2_ particle sample arrangement in the ultrasound experiment; (b) A 2D sectional view of the computational domain and the meshes for the model, including the DI-water, a bubble and a solid particle; (c) an enlarged view of the meshes near the bubble and particle, i.e., the c region in (b); (d) a schematic illustration of the solid particle in contact with the bubble.

Fig.2a shows the sample arrangement for the bubble-particle interaction experiment. The same quartz tube as that in Fig. 1a was used. The solid particles used were Molybdenum disulfide (MoS_2_)particles (Sigma-Aldrich 69860-100G, 6 ∼ 40 μm), which were initially contained in a smaller inner quartz tube holder inside the bigger quartz tube before ultrasound processing as illustrated in Fig. 2a (more clearly in the enlarged view on the right). The modelling is simplified version of such experiment by only considering one single MoS_2_ particle. Fig. 2b and c show the corresponding domains and meshes. Refined unstructured meshes were used around the bubbles as shown in Fig. 2c.

#### 2.3.3 The bubble-particle contact angle and materials properties

To account for the solid surface properties, i.e., hydrophobic or hydrophilic properties, the contact angle, *θ_d_*, is introduced as shown in Fig. 2d. In the range of 90°≤ *θ_d_*, ≤ 120°, it represents hydrophobic; in the range of 10° ≤ *θ_d_*, ≤ 90°, it represents hydrophilic.

The physical properties of the two particles used in this study are listed in Table 4. When a particle moves while in touch with the gas–liquid interface, the dynamic contact angle $\theta_{d}$ also moves as shown in Fig. 2d. The empirical formula proposed by Kistler et al. [58]is used to calculate the dynamic contact angle during the wetting process, it is written as.

(18) $$\theta_{d}=f_{H}\left(Ca+f_{H}^{-1}\left(\theta_{e}\right)\right)$$

where $f_{H}\left(x\right)$ is the Hoffman function [59], which can be expressed as:

(19) $$f_{H}\left(x\right)=\arccos\left\{1-2\tanh\left[5.16{\left(\frac{x}{1+1.31x^{0.99}}\right)}^{0.706}\right]\right\}$$

**Table 4:** Properties used for bubble-particle interaction simulation.

Parameters	Symbol & unit	Ge [61]	MoS_2_[62]
Density	$\rho_{p}\left(\mathrm{kg}.m^{-3}\right)$	5.323	5.06
Contact angle	$\theta_{d}\left({}^{\circ}\right)$	65	91.6

$\mathrm{Ca}=\mu_{l}V_{\mathrm{CL}}/\sigma$ is the capillary number [60].$V_{\mathrm{CL}}$ is the speed of the TPCL, which is calculated by:

(20) $$V_{\mathrm{CL}}=\left(U-u\right)∙\eta$$

$\theta_{e}$ is the static contact angle and takes different values depending on the direction of the TPCL motion.

## 3 Simulation case studies and discussion

In this section, we give a number of simulation cases and demonstrate how we used the X-ray images to validate the models and the simulation results, including a single bubble oscillation and implosion. Based on the validated models, we are able to predict the interactions between bubbles and bulk or particle materials with different properties.

### 3.1 Bubble dynamics modelling and validation

**Fig.3:**
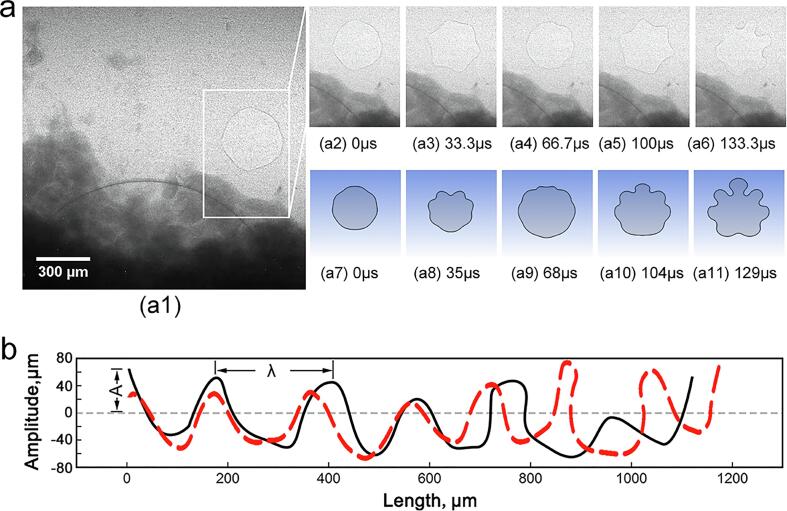
Simulation and validation of a single bubble dynamic movement, (a) during quasi-static oscillation in water containing hydrophobic MoS_2_ particles captuted at 37,000 fps, showing the surface wave development at the bubble boundary due to Raleigh-Taylor instability (a1-a6). The simulated results (a7-a11) are shown below the corresponding X-ray images for comparison. (b) Comparison of the wavelength (along the circumferential direction) and amplitude (along the radial direction) of the distorted bubble boundary between the X-ray imaged bubble (Fig. 3a6) and simulated bubble (Fig. 3a11). (More vivid X-ray imaging and simulation results are presented in Video 1 and Video 2, respectively).

To validate the accuracy and correction of compressibility of the modelled bubbles in present work, the results from the numerical modelling and the X-ray imagines were compared. MoS_2_ particles (99 % purity with size of 6 ∼ 10 μm) and a specially-designed quartz sample holder were used in this experiment. The detailed experiment set-up, X-ray imaging parameters as well as the synchronization details are the same as described in [26]. The framed region in Fig. 3a (marked by a white rectangle) contains an oscillating bubble only. The simulated quasi-static bubble oscillation and the bubble surface instability are shown below the corresponding X-ray images in Fig. 3a. The simulated results matched the experimental results very well. For instance, the bubble boundary (Fig. 3a6), which has a wavelength of 167 ± 18 m and an amplitude of 40.5 ± 7 m, developed significant distortions as a result of surface instability, as shown in Fig. 3b. They are a 94.9 % agreement between simulation and experiment, indicating that the modelling capture the underlying physics, and the simulation results can be used with sufficient confidence to assist the interpretation of the experimental results.

### 3.2 Single bubble oscillation and implosion

**Fig. 4:**
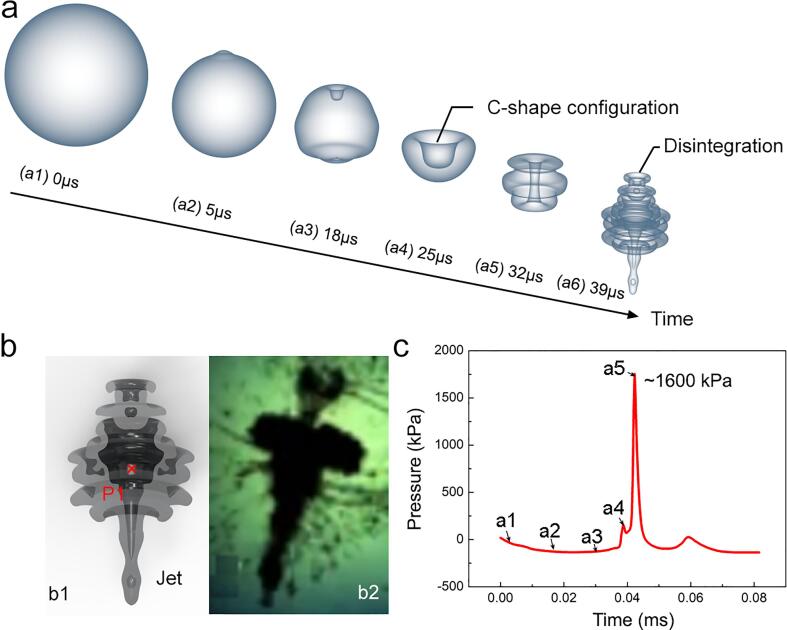
(a) An simulated image sequence, showing the imploding process of a bubble in the compression period of the acoustic cycle; (b) The cross-sectional of (a6) and the optical results in [64] for comparison. (c) The pressure evolution and spike at point P1 (marked with an xin b1) during the imploding process.

Fig. 4a shows the evolution of a single bubble immediately below the sonotrode tip inside the DI-water. The simulation results indicated that the bubbles have a high probability of collapsing and disintegrating in one cycle (∼33 µs), which is consistent with the experimental results we observed before [24]. In this work, four typical stages occurred, namely, the bubble boundary instability (Fig. 4a2-a3), the formation of the C-shape configuration (Fig. 4a4) at top surface of bubbles, the formation of toruses (Fig. 4a5) and bubble disintegration (Fig. 4a6). Simulation results illustrate how the C-shape evolves until the total disintegration. As the C-shape is produced (Fig. 4a3) and further developed(Fig. 4a4) The surface around the C-shape is dragged and distored further into multi-fold toruses configuration. The internal shape of the cavity is quite complex as shown in Fig. 4b1. At this stage, it is just at the brink of a total disintegration, i.e., implosion. The simulation result is consistent with previous experimental results [64] as shown in Fig. 4b2. We also plotted the pressure profile at the center of bubble disintegration, marked as P1 in Fig. 4b1. Its shock wave pressure peak exceeded ∼1600 kPa, which agrees with our earlier measurement [23]. The pressure is one order of magnitude greater than the acoustic pressure around the bubble (∼150 kPa).

### 3.3 Bubble coalescence

**Fig. 5:**
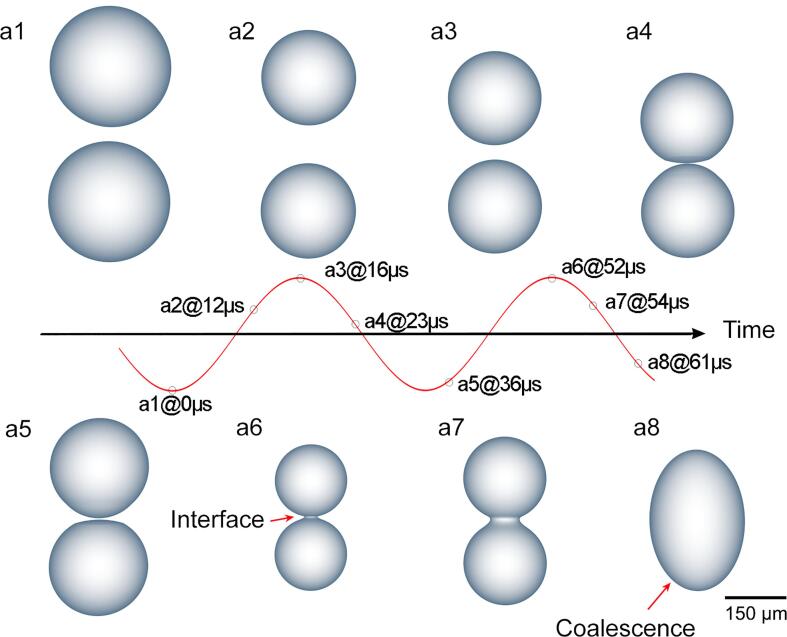
Two bubbles coalescence dynamics in two ultrasonic periods at simulated time steps of (a1) 0 s, (a2) 12 s, (a3) 16 s, (a4) 23 s, (a5) 36 s, (a6) 52 s, (a7) 54 s, and (a8) 61 s, respectively.

Our experiment observation has showed that, at an appropriate distance away from the sonotrode tip, quasi-static bubble oscillation and coalescence often occur in many thousands of ultasound cycles. Based on those observations, we did corresponding simulation as shown in Fig. 5 to understand the effect of pressure wave on the bubble coalescence dynamics. In DI-water, two bubbles (with the same initial radii of ∼150 µm) oscillated and approached to each other. As they moved closer and then touched at one point (Fig. 5a1-a5), the contacted surfaces became flattened and widened, with a thin liquid film trapped in between (Fig. 5a4-a5). As the bubbles get even closer, the two bubbles begin to join together (Fig. 5a6-a7). Although the phenomenon is similar to the coalescence of two droplets in a static condition as reported in Ref. [27]. In an ultrasound field, theoscillating pressure field indeed plays a decisive role in controlling bubble coalescence dynamics.For example, for two touching bubbles, their coalescence can be accelerated in the compression part of the acoustic cycle, if the two bubbles can complete the liquid film drainage and film rupture process in that half cycle. Otherwise if they cannot complete the process, then at the next rarefaction half cycle, the pressure can simply "pull" the two bubbles apart, either delaying or completely alter any subsequent possible coalescence process. The coalescence of the two bubbles was completed at the 61 µs as shown in Fig. 5a8. In terms of coalescence time, Lebon et al. [65] reported that at a short distance (less than 1 mm), bubbles coalesce in a few acoustic cycles [66] which is consistent with our simulation.

### 3.4 Bubble interaction with graphite bulk material

**Fig. 6:**
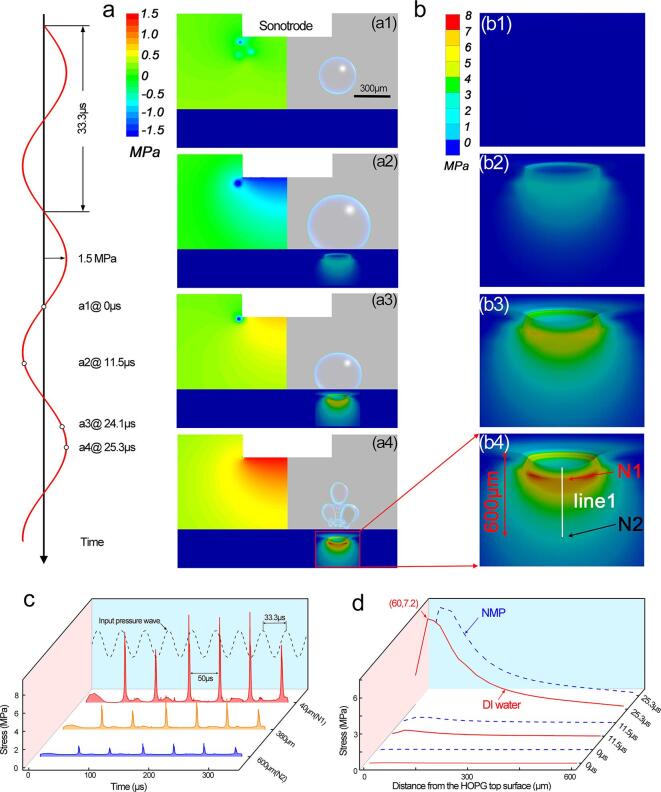
(a1-a4) The simulated pressure contour maps in DI water produced by the vibrating sonotrode (a frequency of 30 kHz and an input pressure amplitude of 1.5 MPa as indicated on the left-hand side) in one ultrasound period at the simulation time step of (a1) 0 μs, (a2) 11.5 μs, (a3) 24.1 μs, and (a4) 25.3 μs, respectively. The simulated typical bubble (based on the individual bubble dynamics shown in Fig. 5a) oscillation and implosion behaviours are displayed alongside (on the right) with the pressure contour on top of HOPG. (b) shows the time evolved stress (b1)-(b4) distributions in HOPG materials induced by the single bubble implosion, more vivid dynamic information can be seen in Video 3. (c) Shows the impulsive stress profiles in 10 ultrasound cycles due to bubble implosion (data were extracted at 40 μm, 360 μm and 600 μm below the HOPG top surface as indicated by line 1 in Fig. 6b4. (d) In a single ultrasound cycle, the time-evolved stress profiles along line 1 in Fig. 6b1-b4 in DI water and NMP.

In this case, we use the HOPG as the bulk material. Firstly, the acoustic pressure distribution inside the liquid was simulated and shown in Fig. 6a. With an input peak pressure of 1.5 MPa at the sonotrode tip, the acoustic pressure reached the HOPG top surface was less than ∼ 1 MPa in DI water (the distance between the sonotrode tip and the HOPG top surface was 880 µm). Secondly, a bubble seed with an initial radius of 150 µm was “planted” into the pressure field 250 µm above the HOPG top surface. Its oscillation and implosion behaviors were simulated and displayed on the right-hand side of Fig. 6a. Fig. 6b shows that the bubble implosion induced impulsive stresses, respectively, immediately below the HOPG surface. At bubble implosion, the impulsive stress propagated ∼ 600 µm deep into the HOPG and reached a peak value of ∼7.28 MPa at the HOPG surface. Its peak values emerged on the surface of HOPG and dramatically decreased with depth. More importantly, under the cyclic ultrasound pressure field, bubble implosions and the resulting impulsive stresses occurred cyclically at the surface as typically indicated in Fig. 6c. In previous studies, Alaferdov et al. [67] also used a low power ultrasound bath (100 W, 37 kHz) to make graphite nanoflakes from natural graphite powders of 1–3 mm. They suggested that the shock waves and microjet flow produced by the cavitation bubbles collapsed near the graphite flake surface were big enough to break the graphite polycrystals. The pressure required to separate two graphene sheets is estimated to be 7.2 MPa, which is almost consistent with our findings. The bubble oscillated and then imploded at the HOPG top surface in less than one ultrasound cycle (∼33.3 µs). The distribution of the stresses created by bubble implosion are displayed in Fig. 6b-c. More importantly, under the cyclic ultrasound pressure field, bubble implosions and the resulting impulsive stresses occurred cyclically at the surface as typically indicated in Fig. 6c. The gap between two consecutive stress pulses was calculated as ∼50 μs, about ∼17 μs longer than one ultrasound period. Yusuf, et al. [68] provided in-situ high-speed cavitation measurements, the results shown that all general oscillations (non-deflating collapses and collapses) occur on a 50 μs timescale, which is almost exactly the same as our predict results. Clearly, such cyclic impulsive stresses imposed a fatigue behavior at the HOPG top surface. This model is useful for evaluating a variety of application scenarios. For example, it may quantify stress intensity and duration as a function of exfoliation length in ultrasonic-assisted liquid phase exfoliation process. DI water and NMP are two typical solvents used in liquid phase exfoliation. Therefore, we examined two different liquids and the results showed that the difference in stress caused by a single bubble was not substantial, as shown in Fig. 6d. This prediction indicated the prospect and potential for the widespread use of DI water instead of NMP in sustainable and industrially scalable ultrasonic liquid phase exfoliation process.

### 3.5 Interaction between a bubble and particles with different surface properties

**Fig. 7:**
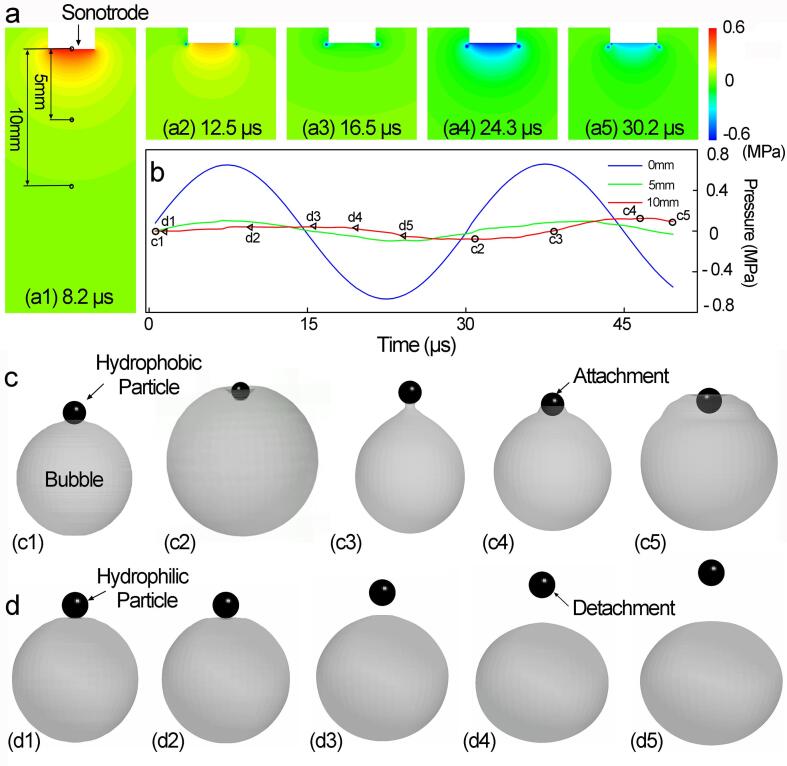
(a) and (b) The simulated pressure contour maps within one ultrasound cycle at 8.2 μs, 12 μs, 16.7 μs, 24.3 μs as well as 30.2 μs and the corresponding pressure profiles at 0 mm,5 mm and 10 mm below the sonotrode tip on the µs scale. Two typical bubble-particle dynamic interaction cases are presented in (c)-(d): (c) an oscillating bubble interacts with a hydrophobic particle at 10 mm below the sonotrode tip; (d) an oscillating bubble interacts with a hydrophilic particle. The corresponding pressure and time for the bubble-particles interaction in Fig. 7c-d are shown in Fig. 7b. (More vivid simulation results are presented in Video 4 and Video 5, respectively).

To fully understand quantitively the bubble–particle interaction dynamics in the ultrasound field, we also simulated the complex and coupled bubble–fluid–solid interaction. Here, we use particles with different surface properties, e.g., hydrophobic, hydrophilic, etc, as the model materials to study the interaction of oscillating bubbles and particles. Fig. 7a illustrate the simulated acoustic pressure field based on the measured sonotrode vibration amplitude (i.e. the pressure input). Two representative locations, i.e. 5 mm and 10 mm below the sonotrode tip were selected for simulating bubble implosion and bubble oscillation as well as the dynamic interaction between the particles and the imploding and oscillating bubbles.

Fig. 7c shows the case of a hydrophobic particle. the particle initially attached to the bubble surface was “glued” onto the bubble boundary because of the hydrophobic effect. As the bubble continued to oscillate and at the bubble contraction stage, the drag force from the hydrophobic particle elongated the bubble into a pear-shape (Fig. 7c3, c4). More interestingly, at the bubble expansion stage, the enlarged and expanded bubble surface started to wrap around the particle (Fig. 7c4, c5) which is possible to trap the particle inside the bubble. Fig. 7d shows the case of a hydrophilic particle. The particle that initially touched the bubble boundary was transported away from the oscillating bubble by the fluid flow and no more interaction occurred. Hydrophilic particles are hardly seen to stay at the bubble surface. Such phenomena have been frequently observed in our previous work when studied the Ge particles interaction with cavitation bubbles.

## 4 Conclusion

In this paper, the VOF and CSF methods were used to develop abubble dynamic model for the simulation of bubble oscillation and implosion behavior. Ultrafast synchrotron X-ray imaging was used to collect image data for validating the model. Coupled bubble-bulk materials interaction and bubble-particle interaction simulations were also made based on the validated model. The important findings of this research are:

(i) In an alternating acoustic pressure field, surface instability is developed at the bubble boundary in the process of oscillation. Once the surface instability exceeds the stable amplitude, bubble implosion occurs, creating shock wave and highly deformed, irregular bubble boundaries as well as many small bubble fragments.
(ii) In bubble-bulk material interaction, ultrasonic bubble implosion can produce cyclic impulsive stresses with a peak value of up to ∼ 7.28 MPa into the HOPG materials, propagating ∼ 600 µm deep into the graphite, resulting in µs fatigue exfoliation of graphite layers.
(iii) In bubble-particle interaction, hydrophobic particles tend to attach to the bubble boundary due to the adsorption force. The drag force from the hydrophobic particle resulted in asymmetric shape development for the bubble which is the possible underlying mechanism for particle wrapping to occur. While the hydrophilic particles do not have energy favourable condition to attach onto the bubble surface, and they are often carried away from the bubbles by the moving liquid flow.

## CRediT authorship contribution statement

**Ling Qin:** Conceptualization, Data curation, Methodology, Software, Investigation, Validation, Visualization, Formal analysis, Writing – original draft. **Kyriakos Porfyrakis:** Writing – review & editing, Funding acquisition. **Iakovos Tzanakis:** Writing – review & editing, Funding acquisition. **Nicole Grobert:** Writing – review & editing, Funding acquisition. **Dmitry G. Eskin:** Writing – review & editing, Funding acquisition. **Kamel Fezzaa:** Investigation, Writing – review & editing. **Jiawei Mi:** Conceptualization, Methodology, Funding acquisition, Project administration, Resources, Supervision, Data curation, Investigation, Writing – review & editing.

## Declaration of Competing Interest

The authors declare that they have no known competing financial interests or personal relationships that could have appeared to influence the work reported in this paper.

## Data Availability

Data will be made available on request.
